# Exposure to Nickel Oxide Nanoparticles Induces Acute and Chronic Inflammatory Responses in Rat Lungs and Perturbs the Lung Microbiome

**DOI:** 10.3390/ijerph19010522

**Published:** 2022-01-04

**Authors:** Mi-Jin Jeong, Soyeon Jeon, Hak-Sun Yu, Wan-Seob Cho, Seungho Lee, Dongmug Kang, Youngki Kim, Yoon-Ji Kim, Se-Yeong Kim

**Affiliations:** 1 Department of Parasitology and Tropical Medicine, Medical College, Pusan National University, Yangsan 50612, Korea; mijin95@pusan.ac.kr (M.-J.J.); hsyu@pusan.ac.kr (H.-S.Y.); 2 Lab of Toxicology, Department of Health Sciences, The Graduate School of Dong-A University, Busan 49315, Korea; jsy0418@donga.ac.kr (S.J.); wcho@dau.ac.kr (W.-S.C.); 3 Research Institute for Convergence of Biomedical Science and Technology, Pusan National University Yangsan Hospital, Yangsan 50612, Korea; 4 Department of Occupational and Environmental Medicine, Pusan National University Yangsan Hospital, Yangsan 50612, Korea; cjsfhleo10@pusan.ac.kr (S.L.); kangdm@pusan.ac.kr (D.K.); mungis@pusan.ac.kr (Y.K.); 5 Environmental Health Center of Asbestos, Pusan National University Yangsan Hospital, Yangsan 50612, Korea; harrypotter79@pusan.ac.kr; 6 Department of Preventive and Occupational & Environmental Medicine, Medical College, Pusan National University, Yangsan 50612, Korea

**Keywords:** nickel oxide nanoparticles, lung microbiome, inflammatory response

## Abstract

Nickel oxide nanoparticles (NiO NPs) are highly redox active nanoparticles. They can cause acute and chronic inflammation in rat lungs. Unlike the gut microbiome, the association between the lung microbiome’s role and pulmonary inflammatory response to inhaled nanoparticles remains largely unexplored. We aimed to explore the interaction between the lung microbiome and inflammatory responses in rats exposed to NiO NPs. Thirty female Wistar rats were randomly categorized into control and low- (50 cm^2^/rat), and high- (150 cm^2^/rat) dose NiO NPs exposure groups. NiO NPs were intratracheally instilled, and cytological, biochemical, proinflammatory cytokine, and lung microbiome analyses of bronchoalveolar lavage fluid were performed at 1 day and 4 weeks after instillation. NiO NPs caused a neutrophilic and lymphocytic inflammatory response in rat lung. We demonstrated that exposure to NiO NPs can alter the lung microbial composition in rats. In particular, we found that more *Burkholderiales* are present in the NiO NPs exposure groups than in the control group at 1 day after instillation. Dysbiosis in the lung microbiome is thought to be associated with acute lung inflammation. We also suggested that *Burkholderiales* may be a key biomarker associated with lung neutrophilic inflammation after NiO NPs exposure.

## 1. Introduction

As various metallic and metal oxide nanoparticles, such as silver, gold, zinc oxide, copper oxide, lead oxide, and nickel oxide nanoparticles (NiO NPs), are being developed and used, their toxicity in vitro and in vivo has been studied [1,2]. The fate of engineered nanoparticles has not been fully evaluated. Although nanoparticles have the same chemical formula, they show different toxicities and biokinetics depending on physicochemical properties such as size, shape, and crystalline structure [3]. Moreover, inhaled nanoparticles that are small enough (1–100 nm) can reach the lower airways and gain access to the air–blood barrier (0.1–0.2 μm) in lung’s alveoli. Therefore, nanoparticles can freely cross the air–blood barrier owing to its large surface area, following rapid absorption owing to extensive vascularization of the lung [4,5]. According to a previous study, inhaled gold nanoparticles can be deposited in the lungs and other organs, such as the liver, kidney, and blood vessels, through translocation into the systemic circulation [6]. Even the potential for accumulating gold nanoparticles in the brain via the olfactory neural pathway has been reported [7]. Overall, the biokinetics of inhaled nanoparticles after exposure is accumulation in the lung, translocation to secondary organs, and excretion through urine and feces [3,7].

NiO NPs are used as industrially photoelectric and recording materials, catalysts, sensors, and so on. Therefore, they are important in industrial toxicology and occupational health risk management [8]. The degree of pulmonary NiO NP-based toxicology also depends on major factors, such as dose of exposure and biopersistence in the lungs [9,10]. Moreover, NiO NPs do not dissolve well in weakly basic solutions, such as physiological saline or interstitial fluid, but dissolves well in acidic environments, such as lysosomes of alveolar macrophages [3,9,11]. Thus, the pulmonary clearance of inhaled NiO NPs can happen after the phagocytosis of alveolar macrophages, and the burden on the liver or blood is not considered high compared with other nanoparticles owing to the lower solubility [3]. Additionally, because NiO NPs have a higher biopersistence than other nanoparticles in the lung, they can cause chronic inflammation for 1–3 months and can persist in the lungs for up to 6 months [9,12]. Previous studies have reported that exposing NiO NPs to the rat lung causes acute neutrophilic inflammation through activation of NLRP3 inflammasomes and production of reactive oxygen species (ROS) [11,12,13]. NiO NPs can also reside in the lungs for a long time and induce a chronic inflammatory response [12,14], resulting in NiO NPs contributing to the induction of lung delayed-typed hypersensitivity (DTH) responses and pulmonary alveolar proteinosis (PAP) in rats [11,14].

The human microbiome is composed of communities of bacteria, viruses, and fungi that have a greater complexity than that of the human genome itself. The human microbiome plays an important role in controlling homeostatic mechanisms, such as resistance to infection and inflammation [15]. Based on sequence-based techniques such as 16S ribosomal RNA microarrays, it has been reported that lung microbiota may modulate chronic pulmonary inflammation and play a critical role in determining features and severity of diseases. However, microbiome–host interactions in the lung and their mechanical relationship with chronic respiratory diseases remain uninvestigated [16,17,18,19]. To date, no study has reported on the interaction between the pulmonary inflammatory response owing to inhaled nanoparticles and the lung microbiome, such as microbiome–host interactions [4,20]. Previous studies have found that exposure to nanoparticles disturbs the gut microbiome and induces inflammatory responses in the intestine [21,22]. Despite increasing attention to the gut microbiome and exposure to nanoparticles, few studies have examined the lung microbiome’s role in response to inhaled nanoparticles. Therefore, we aimed to explore the interaction between lung microbiome and inflammatory responses in rats exposed to nanoparticles. In this study, we hypothesized that pulmonary exposure to NiO NPs in rats alters the lung microbial composition and induces microbe–nanomaterial interaction.

## 2. Materials and Methods

### 2.1. Physicochemical Characteristics and Analysis Methods of NiO NPs

NiO NPs were purchased from Nanostructured and Amorphous-Materials (Houston, TX, USA) and used in our experiments. The physicochemical analysis of NiO NPs followed previously described methods [11]. The surface area of NiO NPs was measured using a Micromeritics Tristar 3000 analyzer (Micromertitics Ltd., Bedfordshire, UK) from ParticleCIC Ltd. (Leeds, UK). The average size of NiO NPs was determined using transmission electron microscopy (TEM; JEM-1200EXII, JEOL, Tokyo, Japan) and more than 100 individual particle sizes were analyzed using the software. Endotoxin measurements were evaluated using an endpoint chromogenic Limulus amoebocyte lysate assay kit (Cambrex, Walkersville, MD, USA).

### 2.2. Preparation of a NiO NPs Suspension

The suspension of nanoparticles used in our in vivo study was prepared using a previously described method [23]. Briefly, the stock solution of NiO NPs was dissolved in distilled water to a 10-fold concentration for 10 min using a bath sonicator (Saehan Sonic, Seoul, Korea). The stock solution was added to heat-inactivated Wistar rat serum and subjected to sonication for 10 min in a bath sonicator. Lastly, PBS was added according to the working solution concentration (50 and 150 cm^2^/rat) and dispersed for 10 min in the bath sonicator. Nanoparticles must be used in the dispersant because nanoparticles agglomerate in PBS. Thus, heat-inactivated Wistar rat serum was 3% of the working solution and used as a dispersant in this experiment. The control group used PBS containing 3% of heat-inactivated Wistar rat serum.

### 2.3. Intratracheal Instillation of NiO NPs

NiO NPs were intratracheally instilled in rats. Purchased animals were 6-week-old specific-pathogen-free female Wistar rats from Korea Central Experimental Animals. They acclimatized for 2 weeks before experimentation. The rats were maintained at 23 ± 1 °C in an environment with 40–60% humidity. Thirty female Wistar rats were randomly categorized into control group, low-dose (50 cm^2^/rat), and high-dose (150 cm^2^/rat) NiO NPs exposure groups. Intratracheal instillation was performed according to a previously described method [24]. Shortly, rats were anesthetized with isoflurane using a rodent anesthesia system (VetEquip, Pleasanton, CA, USA). The 16-gage polycarbonate catheter was inserted into the trachea of a sufficiently anesthetized rat. Next, the catheter and 1 mL syringe were connected. Lastly, 500 µL of the suspension of NiO NPs and that of the control groups were instilled. The animal experiments in this study were approved by Pusan National University Institutional Animal Care and Use Committee (No. PNU-2019-2364). Animal experiments were conducted while using minimal number of animals to obtain necessary results, and the experimenter tried to minimize the suffering of animals.

### 2.4. Bronchoalveolar Lavage Fluid (BALF) Harvesting and Cytological Analysis

The rats were sacrificed 1 day and 4 weeks post-instillation to analyze lung inflammation patterns. A rodent anesthesia system euthanized the rats, and the inferior vena cava was dissected. The trachea was intubated using a 14-gage stainless needle and then sutured using a thread. A sterile syringe was connected and lavage was performed a total of 4 times using 8 mL PBS. BALF was centrifuged at 2000 rpm for 5 min, and the supernatant of the first lavage fluid was separated for biochemical and cytokine analysis. Cell pellets from four lavage fluids were resuspended in 1 mL of PBS containing 10% FBS. The total number of resuspended cells was measured using Nucleo Counter (Chemometec, Allerod, Denmark). A total of 4 × 10^4^ cells were attached to a glass slide (Marienfeld, Lauda-Königshofen, Germany) at 500 rpm for 5 min using Cytospin (Hanil, Seoul, Korea) and then fixed for 5 min in methanol. The fixed cells were stained using Diff–Quik (Thermo Fisher Scientific, Waltham, MA, USA). A light microscope (Nikon, Tokyo, Japan) was used to measure 300 cells per slide based on the cell type.

### 2.5. Biochemical Analysis of BALF

The levels of LDH, a marker of cell death, were measured in the supernatant of first BALF [25]. Moreover, the levels of total protein and phospholipids as well as turbidity associated with PAP were measured in the supernatant of the first BALF [11,26]. LDH was quantified using the LDH assay kit (Roche Diagnostics; Mannheim, Germany) and total protein was quantified using a bicinchoninic acid (BCA) assay kit (Thermo Fisher Scientific). The turbidity of the BALF was quantified by measuring the absorbance at 600 nm using a microplate reader, and phospholipids were measured using the phospholipid assay kit (Bioassay Systems, Hayward, CA, USA).

### 2.6. Proinflammation Cytokine Analysis of Supernatant of BALF

To determine the mechanism of inflammation by NiO NPs in the lung, the levels of proinflammatory cytokines, including interleukin (IL)-1β, IL-2, IL-4, IL-6, and IL-10, and also cytokine-induced neutrophil chemoattractant-3 (CINC-3), eotaxin, granulocyte-macrophage colony-stimulating factor (GM-CSF), interferon (IFN)-γ, monocyte chemoattractant protein (MCP)-1, and tumor necrosis factor (TNF)-α, were measured in the supernatant of the first BALF. All cytokine kits were purchased from R & D systems (Duoset kits, Minneapolis, MN, USA) and used according to their corresponding protocol. Briefly, 100 μL per well of the captured antibody was added, followed by overnight incubation at room temperature (RT). The wells were then washed 4 times using PBS with tween 0.05% and then blocked using 1% BSA in PBS for 1 h at RT. Subsequently, each standard and BALF was added at a rate of 100 μL per well, following another incubation at RT for 2 h. Subsequently, wells were washed 4 times, after which 100 μL detection antibody was added, and the setup was incubated at RT for 2 h. Next, wells were washed again 4 times, following the addition of 100 μL streptavidin-horseradish peroxidase (HRP) and incubation at RT for 20 min. Lastly, the wells were washed 4 times and then 100 μL of 3,3′,5,5′-tetramethylbenzidine (TMB) was added, following incubation for 15–30 min by blocking light. Finally, the color reaction was stopped using 50 μL of 2 N H_2_SO_4_, and absorbance was measured using a microplate reader at 450 and 570 nm.

### 2.7. Lung Histopathology

Lung tissues were fixed with formaldehyde and embedded in paraffin. The paraffin blocks of lung tissue were cut to obtain 5 μm sections. The sections were deparaffinized in three changes of xylene substitute for 10 min and washed in two changes of absolute alcohol for 5 min each, 95% alcohol for 2 min, 70% alcohol for 2 min, and then in running tap water for 1 min. Samples were stained using a Harris hematoxylin solution for 8 min and washed in running tap water for 5 min. Next, samples were differentiated in 1% acid alcohol for 30 s and washed again. The slides were blued in 0.2% ammonia water or saturated lithium carbonate solution for 30 s to 1 min. The slides were washed in running tap water for 5 min and rinsed in 95% alcohol for 10 dips. The slides were counterstained with an eosin Y solution for 30 s to 1 min and dehydrated briefly using 95% alcohol, followed by complete dehydration in 2 changes of absolute alcohol for 5 min. Samples were cleared in two changes of xylene for 5 min and mounted with a xylene-based mounting medium. Finally, these sections of embedded tissues were stained with hematoxylin and eosin (H&E) examined microscopically.

### 2.8. BALF Microbiome Analysis

Genomic DNA was obtained from the BALF for microbiome analysis. The DNA quantity was analyzed using Victor 2 fluorometry using the pico green (Invitrogen, cat #P7589) method. The starting genomic material was analyzed using fluorescence-based quantification. Owing to RNA and other contaminants commonly found in gDNA preparations, UV spectrometry methods based on 260 OD readings were conducted to roughly estimate the DNA concentration. Gel electrophoresis was conducted to assess the condition of the DNA.

### 2.9. DNA Extraction and Quantification

DNA was extracted using DNeasyPowerSoil Kit (Qiagen, Hilden, Germany) according to the manufacturer’s instructions. The extracted DNA was quantified using Quant-IT PicoGreen (Invitrogen).

### 2.10. Library Construction and Sequencing

The sequencing libraries were prepared according to the Illumina 16S Metagenomic Sequencing Library protocols to amplify the V3 and V4 regions. Two nanograms of input gDNA were PCR-amplified with 1 × reaction buffer, 1 nm dNTP mix, 500 nm each of the universal F/R PCR primer, and 2.5 U of Herculase II fusion DNA polymerase (Agilent Technologies, Santa Clara, CA, USA). The cycling conditions for the 1st PCR were 3 min at 95°C for heat activation and 25 cycles of 30 s at 95 °C, 30 s at 55 °C, and 30 s at 72 °C, followed by a 5 min final extension at 72 °C. The universal primer pair with Illumina adapter overhang sequences used for the first amplifications were V3-F, 5′-TCGTCGGCAGCGTCAGATGTGTATAAGAGACAGCCTACGGGNGGCWGCAG-3′, and V4-R, 5′-GTCTCGTGGGCTCGGAGATGTGTATAAGAGACAGGACTACHVGGTATCTAATCC-3′. The first PCR product was purified using AMPure beads (Agencourt Bioscience, Beverly, MA, USA). Following purification, 2 µL of the first PCR product were PCR-amplified for final library construction containing the index using a NexteraXT Indexed Primer. The cycling conditions for the second PCR were the same as those for the 1st PCR, except for the implementation of 10 cycles. The PCR product was purified using AMPure beads. The final purified product was then quantified using qPCR according to qPCR Quantification Protocol Guide (KAPA Library Quantification kits for IlluminaSequecing platforms) and qualified using the TapeStation D1000 ScreenTape (Agilent Technologies, Waldbronn, Germany). Paired-end (2 × 300 bp) sequencing was performed using Macrogen on the MiSeq™ platform (Illumina, San Diego, CA, USA).

### 2.11. Statistical Analysis

Data were analyzed using the GraphPad Prism software (ver.6.0, LA Jolla, CA, USA) and expressed as mean ± standard deviation (SD). Statistical analysis was performed using one-way analysis of variance (ANOVA) with a post-hoc Tukey’s pairwise comparison test to compare 3 groups; control group, low-dose (50 cm^2^/rat), and high-dose (150 cm^2^/rat) NiO NPs exposure groups. A *p*-value of 0.05 was considered to be statistically significant.

## 3. Results

### 3.1. Physicochemical Characteristics of NiO NPs

The surface area and primary sizes of NiO NPs were 91.8 m^2^/g and 5.3 ± 0.4 nm, respectively. The endotoxins of NiO NPs were not detected, and NiO NPs were well dispersed.

### 3.2. Histologic Changes in the Lung after NiO NPs Instillation

**Figure 1:**
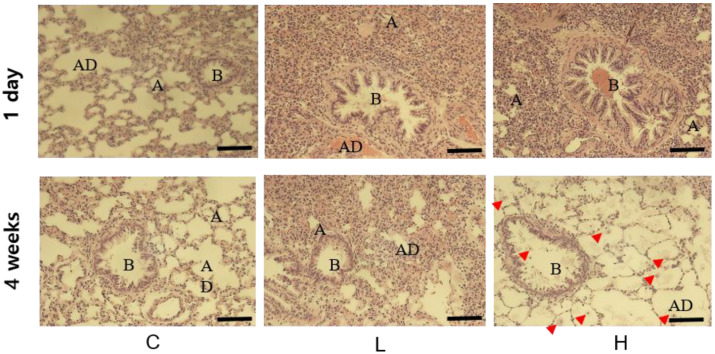
Histological changes in the lungs of rats at 1 day and 4 weeks after instillation of nickel oxide nanoparticles (NiO NPs). Tissues were stained with hematoxylin and eosin. Compared with the control group, the instillation of NiO NPs in rats caused the narrowing of alveolar ducts (AD) and alveoli (A) and blocking of bronchioles (B). In the high-dose group, NiO NPs produced foamy macrophages and exhibited deposition of proteinaceous materials in the alveoli on day 28 after instillation. (Indicator; macrophage, bar = 50 μm). C: control group; L: low-dose group (50 cm^2^/rat); H: high-dose group (150 cm^2^/rat).

Lung histological changes were observed in rats after instillation of NiO NPs using H&E staining. The control groups maintained intact alveolar ducts, alveoli, and bronchioles. One day after instillation, the low- (50 cm^2^/rat) and high-dose groups (150 cm^2^/rat) had narrow alveolar ducts and alveoli and bronchioles were blocked by the infiltration of mononuclear cells compared with the control group. At 4 weeks after instillation, the low- and high-dose groups showed macrophages, immune response cells, and the infiltration of mononuclear cells. In particular, high-dose groups showed foamy macrophages with proteinaceous deposits in the alveoli. Therefore, NiO NPs caused acute and chronic infiltration of inflammatory cells in the lungs and densification secretion of immune cells in rats (Figure 1).

### 3.3. Cytological Analysis of BALF after NiO NPs Instillation

**Figure 2:**
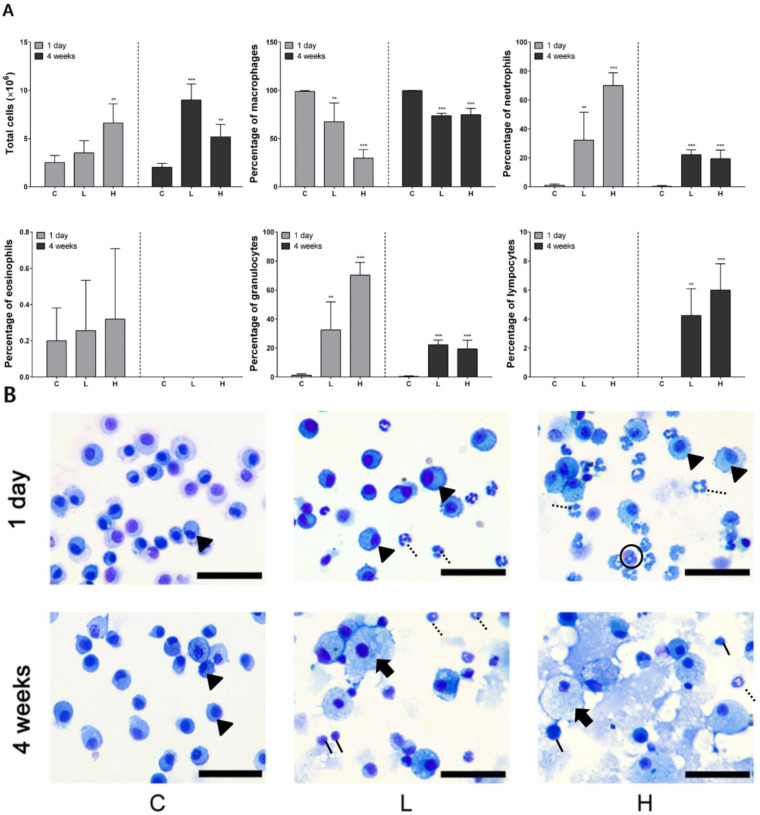
Cytological analysis of bronchoalveolar lavage fluid (BALF) after intratracheal instillation of nickel oxide nanoparticles (NiO NPs). The number of total cells, percentage of macrophages, neutrophils, eosinophils, granulocytes, and lymphocytes is indicated (**A**). Diff–Quik staining images of immune cells in BALF after intratracheal instillation of NiO NPs. Macrophages (arrowhead) were observed after 1 day in C, L, and H. However, foamy macrophages (arrow) were observed only at 4 weeks in the L and H (**B**). Although neutrophils (dotted line) were recruited in the BALF of L and H, lymphocytes (solid line) were recruited at 4 weeks after instillation in BALF of L and H. Eosinophils (circle) were then observed 1 day after instillation in H from the cell image. Data are presented as mean ± standard deviation (SD) of each group. One-way analysis of variance (ANOVA) was performed for the comparison between the NiO NPs-treated and control groups with statistical significance indicated by ** *p* < 0.01 and *** *p* < 0.001. C: control group; L: low-dose group (50 cm^2^/rat); H: high-dose group (150 cm^2^/rat).

One day after instillation, the number of total cells was significantly higher in the high-dose group than in the control group and increased in a dose-dependent manner. The number of neutrophils and granulocytes in the exposed groups significantly increased compared with that in the control group. In the high-dose group, the number of neutrophils was 4.7 × 10^6^ cells/mL, accounting for approximately 70% of the total cells. Conversely, the number of macrophages decreased in a dose-dependent manner compared with that in the control group. Lymphocytes were not detected. At 4 weeks after instillation, the number of total cells significantly increased in the exposed groups and numbers in the low-dose instillation group were higher than those in the high-dose instillation group. The percentages of neutrophils, granulocytes, and lymphocytes in the exposed groups were significantly higher than those in the control group. Macrophages were observed at 1 day in all the groups. However, foamy macrophages, the major PAP marker, were observed at 4 weeks after instillation in the exposed groups. Neutrophils were recruited in BALF of the exposed groups. Moreover, lymphocytes were observed only at 4 weeks after instillation in BALF of the exposed groups. Alternatively, eosinophils were measured only 1 day after instillation in the exposed groups (Figure 2A,B).

### 3.4. Proinflammatory Cytokine Analysis of the BALF Supernatant

**Figure 3:**
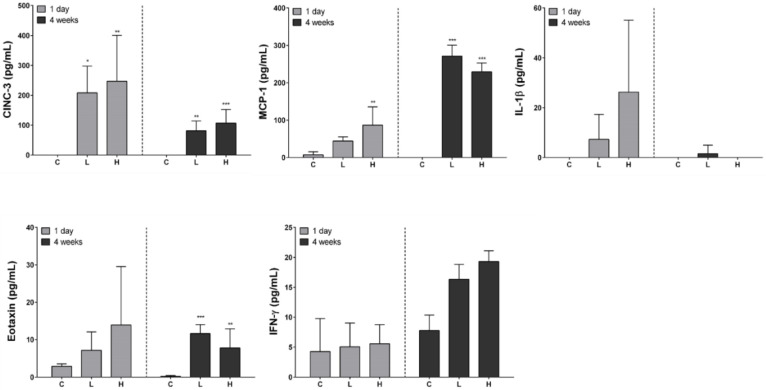
Proinflammatory cytokine analysis of bronchoalveolar lavage fluid (BALF) after intratracheal instillation of nickel oxide nanoparticles (NiO NPs). Levels of cytokine-induced neutrophil chemoattractant-3 (CINC-3), eotaxin, monocyte chemoattractant protein (MCP)-1, interferon (IFN)-γ, and interleukin (IL)-1β are indicated. Data are presented as mean ± standard deviation (SD) of each group. One-way analysis of variance (ANOVA) was performed for the comparison between the NiO NPs-treated and control groups with statistical significance indicated by * *p* < 0.05, ** *p* < 0.01, and *** *p* < 0.001. C: control group; L: low-dose group (50 cm^2^/rat); H: high-dose group (150 cm^2^/rat).

The levels of a total of 11 cytokines (IL-1β, IL-2, IL-4, IL-6, IL-10, CINC-3, eotaxin, GM-CSF, IFN-γ, MCP-1, and TNF-α) in the supernatant of first BALF of all the groups were measured, but IL-2, IL-4, IL-6, IL-10, GM-CSF, and TNF-α were not detected. CINC-3 was increased in a dose-dependent manner at 1 day and 4 weeks after instillation but decreased in a time-dependent manner. Eotaxin levels significantly increased in the exposed groups compared with that in the control group at 4 weeks after instillation. MCP-1 levels increased in a dose-dependent manner 1 day after instillation but did not in a dose-dependent manner at 4 weeks. IFN-γ did not significantly increase at 1 day and 4 weeks after instillation. IL-1 β levels increased in a dose-dependent manner at 1 day but remained undetected at 4 weeks after instillation. Among the detected cytokines, the levels of eotaxin, IFN-γ, and IL-1β were extremely low. The detection limits of eotaxin, IFN-γ, and IL-1β were 7.81, 39.1, and 62.5 pg/mL, respectively (Figure 3).

### 3.5. Biochemical Analysis Results of BALF

**Figure 4:**
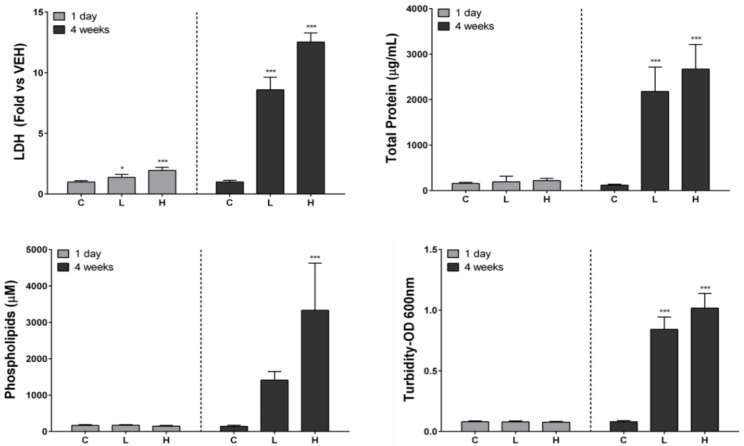
Biochemical analysis of bronchoalveolar lavage fluid (BALF) after intratracheal instillation of nickel oxide nanoparticles (NiO NPs). Lactate dehydrogenase (LDH) levels, total protein levels, turbidity, and phospholipid levels are indicated. Turbidity was measured as optical density at 600 nm. LDH, total protein, phospholipids, and turbidity level in the BALF significantly increased at 4 weeks after instillation. Data are presented as mean ± standard deviation (SD). One-way analysis of variance (ANOVA) was performed for the comparison between the NiO NPs-treated and control groups with statistical significance indicated by * *p* < 0.05 and *** *p* < 0.001. C: control group; L: low-dose group (50 cm^2^/rat); H: high-dose group (150 cm^2^/rat).

Lactate dehydrogenase (LDH) levels significantly increased in a time- and dose-dependent manner at 1 day and 4 weeks after instillation. We measured the levels of total protein, phospholipids, and turbidity associated with PAP in BALF [11,26]. The levels of total protein and turbidity significantly increased in the exposed groups compared with those in the control group. Total protein and turbidity significantly increased in a dose-dependent manner in the exposed groups at 4 weeks after instillation compared with the control group. Proteins and phospholipids accumulated in the rat lung where PAP occurred, and it increased the turbidity of BALF [26]. The levels of phospholipids also significantly increased in the high-dose group at 4 weeks after instillation (Figure 4).

### 3.6. Effect of NiO NPs Exposure on Community Diversity and Richness of the Lung Microbiome in Rats

**Figure 5:**
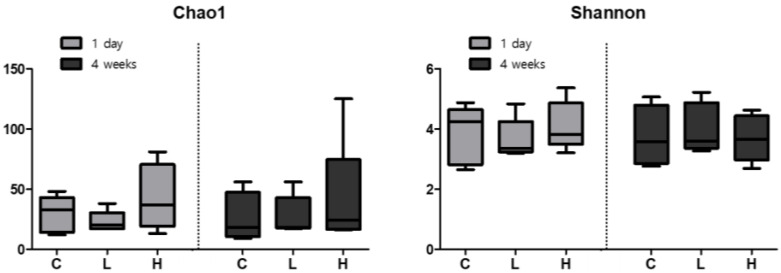
Alpha diversity, which estimates bacterial diversity and species richness. Bacterial diversity (Shannon; right side) and richness estimates (chao1; left) are divided into two groups, reflecting 1 day (light gray) and 4 weeks (dark gray) after intratracheal instillation of nickel oxide nanoparticles (NiO NPs), by using a boxplot. Community diversity and richness were evaluated using the alpha diversity and were not significantly different between exposure groups of NiO NPs and control group. Median values are shown as a line within the box and error bars indicate the standard error of the samples C: control group; L: low-dose group (50 cm^2^/rat); H: high-dose group (150 cm^2^/rat).

Changes of microbiome in the BALF triggered by exposure to NiO NPs were evaluated using DNA extraction. Community diversity and richness were evaluated using the alpha diversity metric. The species richness estimate, Chao1, was not significantly different, but was higher in the high-dose group than in the control group after 1 day and 4 weeks. However, no differences were observed between the control group and the low-dose group. The Shannon index, showing the number and diversity of species, did not show a significant change. In addition, there were no differences in its levels between 1 day and 4 weeks after instillation of NiO NPs (Figure 5).

### 3.7. Effect of the Composition of Lung Microbiome by NiO NPs Exposure in Rats

**Figure 6:**
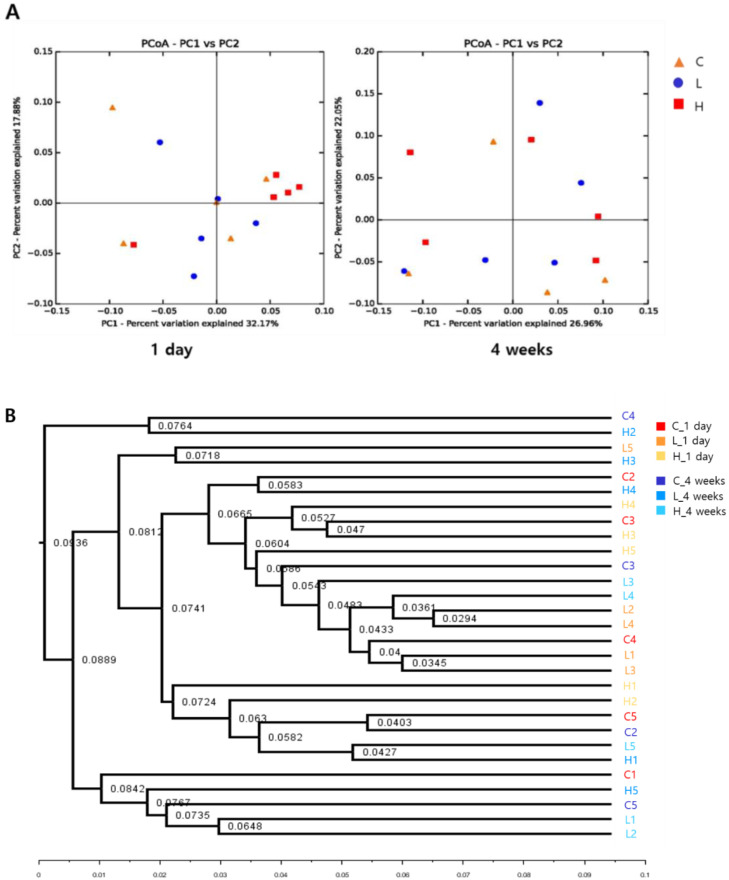
Beta diversity of principal coordinates analysis (PCoA) of weighted unifrac distances (**A**). Unweighted pair group method with arithmetic mean (UPGMA) cluster analysis based on community composition of each sample in at phylum level (**B**). The similarities of lung microbial composition between instillation groups of NiO NPs and control group were not significant at the phylum level. C: control group; L: low-dose group (50 cm^2^/rat); H: high-dose group (150 cm^2^/rat).

The principal coordinates analysis (PCoA) plot is a statistical method used to explore the relative similarities and differences between individual samples. The NiO NPs-exposed groups were not successfully separated. One day after instillation, all groups were separated, with 32.17% and 17.88% variation explained by the PC1 and PC2 principal components, respectively. Similarly, all groups after 4 weeks were divided into 26.96% and 22.05% variation groups explained by the PC1 and PC2 principal components, respectively (Figure 6A). The unweighted pair group method with arithmetic mean (UPGMA) method constructs a rooted tree that reflects the structure present in a pairwise similarity matrix. At each stage, the two closest clusters are combined into a higher-level cluster. This cluster analysis demonstrated that the similarities of lung microbial composition between instillation groups of NiO NPs and control group were not significant in the phylum level (Figure 6B).

### 3.8. Taxonomic Assignment on NiO NP Exposure of the BALF Microbiome

**Figure 7:**
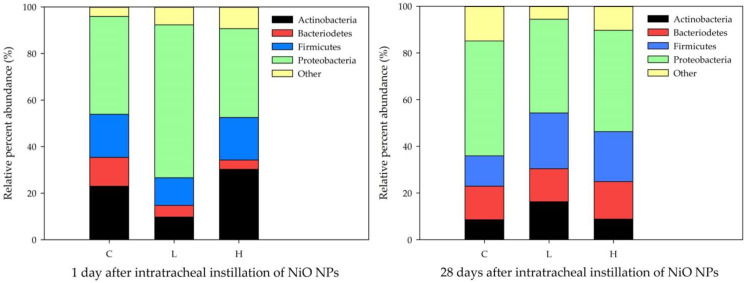
Bacterial taxonomic composition analysis after exposure to nickel oxide nanoparticles (NiO NPs). Bacterial taxonomic assignment was at the phylum level and samples were compared according to NiO NPs concentrations at 1 day and 4 weeks after instillation. NiO NPs induced the change in lung microbial composition in rats at 1 day after instillation. C: control group; L: low-dose group (50 cm^2^/rat); H: high-dose group (150 cm^2^/rat).

Taxonomic statistics show the bacterial assignment for each sample. At the phylum level, *proteobacteria* accounted for a large proportion of all samples. NiO NPs induced the change in lung microbial composition in rats at 1 day after instillation. The proportion of *actinobacteria* increased in the high-dose group (30.23%) compared with that in the control group (23.40%) at 1 day after instillation (Figure 7).

The linear discriminant analysis effect size (LEfSe) determines the features (organisms, clades, operational taxonomic units, genes, or functions) most expected to explain the differences between classes by coupling standard tests for statistical significance with additional tests encoding biological consistency and effect relevance [27]. The LEfSe analysis was performed to compare the control and NiO NPs-exposed groups (Table 1). In the control group, family *Enterococcaceae*, genus *Methylorubrum*, and species *Methylorubrum populi* were more significantly enriched 1 day after instillation at the family, genus, and species levels, respectively. However, in the NiO NPs-exposed group, class *Betaproteobacteria* and order *Burkholderiales* were more significantly enriched at the class and order levels, respectively. Therefore, we observed that the order *Burkholderiales* were enriched in the NiO NPs-exposed groups during acute inflammatory responses. On the 28th day after instillation, however, family *Sutterellaceae* and genus *Sutterella* were more significantly abundant in the control group at the family and genus levels, respectively.

## 4. Discussion

NiO NPs are known to cause various acute and chronic inflammatory responses in the lung. One of the acute inflammatory responses of NiO NPs in the lung is neutrophilic inflammation. Previous animal studies reported acute neutrophilic or eosinophilic pulmonary inflammation with the instillation of NiO NPs in the lung [28,29]. NiO NPs induced persistent inflammation as well as increased the secretion of the proinflammatory cytokines such as IL-1β and MCP-1 and tissue infiltration of macrophages and neutrophils [30]. In our study, the percentages of neutrophils in the NiO NPs-exposed groups significantly increased compared with those in the control group. In the low-dose group (50 cm^2^/rat concentration), it was 32%, and in the high-dose group (150 cm^2^/rat concentration), it was 70% of the total cells at 1 day after instillation. The levels of the proinflammatory cytokines CINC-3 and MCP-1 significantly increased in the NiO NPs-exposed groups compared with those in the control group at 1 day after instillation. CINCs (CXCL-1 (CINC-1), CXCL-2 (CINC-3) and CXCL-3 (CINC-2αβ)) have been established as important neutrophil chemoattractants during lung inflammation [31,32]. MCP-1 also plays an important role in recruiting neutrophils and monocytes/macrophages [31,33]. A neutrophilic inflammatory response of nanoparticles was associated with surface reactivity, such as ROS generation. Nanoparticle-mediated ROS generation is known as one of the main mechanisms related to inflammation in the lung [34,35,36].

NiO NPs are also known as heptene metal particles, which cause delayed hypersensitivity reactions in the skin and lungs. Delayed-type hypersensitivity reactions indicate that macrophages are activated by the cytokine secretion of sensitized Th1 cells. Delayed-type hypersensitivity reactions occur when the skin or lungs are exposed to NiO NPs [11,37]. The chronic inflammatory response of NiO NPs reportedly causes alveolar proteinosis, and neutrophils and lymphocytes are also detected in the lungs [11,36,38]. The causes of PAP due to the inhalation of particles are alveolar type 2 cell proliferation or removal and impairment of surfactant-removal abilities by dead alveolar macrophages. The main markers of alveolar proteinosis are the increased levels of phospholipids and foamy macrophages. In our previous studies, the NiO NPs induced PAP [11,35,39]. The present study also showed a similar pattern, that phospholipids and foamy macrophages increased in the BALF. Moreover, MCP-1 levels associated with macrophage stimulation increased at 4 weeks after instillation. This study found significantly increased levels of phospholipids at 4 weeks after instillation in the high-dose group. The levels of total protein and turbidity significantly increased in the NiO NPs-exposed groups compared with those in the control group.

In general, the lung microbiome of healthy mice is highly variable and influenced and clustered by environmental factors [40]. In this study, we demonstrated that NiO NPs exposure in rats altered the lung microbial composition in the acute inflammatory response phase. The microbial composition in healthy rat lungs was enriched in the phyla *proteobacteria*, *actinobacteria*, *firmicutes* and *bacteroidetes*. This result is similar to that of a study in which rats were exposed to particulate ambient matter [41]. We found no significant difference according to the exposure of NiO NPs at 4 weeks after instillation in the phylum distribution, but the levels of *actinobacteria* increased significantly in the high-dose group compared with those in the control group at 1 day after instillation. There were no significant differences in the alpha diversity and beta diversity analyses of the rat lung microbiome after exposure to NiO NPs. Although it was not significant, the species richness estimate, Chao1, was higher in the high-dose group than it was in the control group. These results were consistent with mice experiments performed using fine particulate matter (PM 2.5) [42]. Dysbiosis in the lung microbiome is associated with lung inflammation and disease, but the directionality of causation remains uncertain [4,43,44]. A study showed that bacteria such as *Mycobacterium tuberculosis* cause chronic inflammation in the lungs, leading to subsequent lung cancer onset [45]. Several studies also showed that some microbes could induce lung cancer by upregulating the ERK and phosphoinositide 3-kinase (PI3K) signaling pathway or pol (ADP-ribose) polymerase 1 (PARP1) [46,47]. Currently, the understanding of the mechanical interaction between inhaled nanoparticles and lung microbiome is limited. The interaction between nanoparticles and the lung microbiome occurs at the interface between the microbial cell surface and nanoparticles. In this interaction, electrostatic forces or hydrogen bonds play an important role in the attachment of nanoparticles to the microbial cell surface. Although the exact mechanism underlying the proinflammatory response that occurs after nanoparticle attachment to microbes remains unclear, it could induce a damaging immune response by rupturing microbial cells, changing the membrane potential, releasing ions at the microbial surface and generating ROS. These interactions between nanoparticles and microbes are important because they influence the host–microbe–environment interplay [4].

We also found the order *Burkholderiales* to be more significantly enriched in the NiO NPs-exposed groups based on LEfSe analysis at 1 day after instillation. *Burkholderiales* are clinically associated with the exacerbation of patients with cystic fibrosis and abundantly found in patients with lung transplantation [44,48,49]. Previous studies have shown that *burkholdeira* spp., included in the order *Burkholderiales*, exert proinflammatory effects on the lung by inducing respiratory epithelial cells to secrete cytokines such as IL-1β, IL-6, IL-8, TNF-α, MCP-1, and chemokine ligand 20 (CCL20) in humans [50,51,52]. In our experiment, although the detected levels of IL-1β were extremely low and not significantly different across groups, IL-1β and MCP-1 levels were higher in the NiO NPs-exposed groups than in the control group, but IL-6 and TNF-α levels were not. IL-8 plays an important role in *burkholderiales cepacia* complex (BCC)-induced exacerbation in patients with cystic fibrosis and is a chemotactic factor for neutrophils [50,51]. Our study showed acute neutrophilic inflammation. The concentrations of neutrophils, CINC-3, and MCP-1 in the NiO NPs-exposed groups were higher than those detected in the control group at 1 day after instillation. Therefore, we suggested that the order *Burkholderiales* may play an important role in neutrophilic lung inflammation after NiO NPs exposure. However, additional studies are warranted to investigate critical species and the mechanical relationship between NiO NPs exposure and neutrophilic inflammation.

Regarding the limitation of this study, we did not conduct the biokinetic analysis of NiO NPs in rat lungs after intratracheal instillation, because our main objective was to explore alterations in the microbiome of the rat lung after exposure to NiO NPs. Other studies on the toxicity of NiO NPs have also dealt with biokinetic-related topics, such as biopersistence and comparison of the levels of retained NiO NPs in lungs according to administration methods, and these topics are clinically important [9,10]. Therefore, it is important to perform quantitative analysis (for example, inductively coupled plasma-mass spectrometry) of Ni’s biokinetics as well after instillation of nanoparticles in future research similar to that in previous studies [6,7]. Whether the results of the experiments on rat lung microbiome can be applied to humans is still an ongoing issue. In the case of the intestinal microbiome, which was previously studied, only 4% of bacterial genes were similar between mice and humans. In addition, environmental factors such as breeding facilities and diet greatly affect the composition of the intestinal microbiome even in the same mouse [52]. This will likely apply to the lungs, which are consistently exposed to external microbes by inhalation and aspiration [40,53]. Therefore, further research is warranted to explore the detailed mechanisms and translate the animal results to humans.

## 5. Conclusions

We demonstrated that exposure of NiO NPs can alter the microbial composition in rat lungs in the acute inflammatory stage (1 day after NiO NPs instillation). Defining the composition of the microbiome and how microbial communities change with exposure may help to explain their role in responding to NiO NPs. This study also proposed the order *Burkholderiales* as a key potential biomarker related to lung neutrophilic inflammation after exposure to NiO NPs.

## Figures and Tables

**Table 1:** Identification of significant differences in bacterial distribution between the nickel oxide nanoparticles (NiO NPs)-exposed (low- and high-dose groups) and control groups based on linear discriminant analysis effect size (LEfSe) analysis.

Day	Phylum	Class	Order	Family	Genus	Species
1 day	*Proteobacteria*	*Alphaproteobacteria*	*Rhizobiales*	*Methylobacteriaceae*	***Methylorubrum***	***Methylorubrum_populi***
		***Betaproteobacteria*** **^†^**	***Burkholderiales*** **^†^**			
	*Firmicutes*	*Bacilli*	*Lactobacillales*	***Enterococcaceae***		
4 weeks	*Proteobacteria*	*Betaproteobacteria*	*Burkholderiales*	***Sutterellaceae***	***Sutterella***	

Order *burkholderiales* were enriched in the NiO NPs-exposed group compared with the control group at 1 day after instillation. The levels of terms in bold were statistically different between the NiO NPs-exposed and control groups by LEfSe. ^†^ Higher in the NiO NPs-exposed group than it was in the control group.

## Data Availability

Not applicable.
